# Uncovering the Horseshoe Effect in Microbial Analyses

**DOI:** 10.1128/mSystems.00166-16

**Published:** 2017-02-21

**Authors:** James T. Morton, Liam Toran, Anna Edlund, Jessica L. Metcalf, Christian Lauber, Rob Knight

**Affiliations:** a Department of Computer Science, University of California, San Diego, La Jolla, California, USA; b Department of Pediatrics, University of California, San Diego, La Jolla, California, USA; c Department of Mathematics, École Normale Supérieure de Lyon, Lyon, France; d Genomic Medicine Group, J. Craig Venter Institute, La Jolla, California, USA; e Department of Animal Sciences, Colorado State University, Fort Collins, Colorado, USA; f Nestle Institute of Health Sciences, Lausanne, Switzerland; Pacific Northwest National Laboratory

**Keywords:** decomposition, horseshoe, microbial ecology, pH, soil

## Abstract

The horseshoe effect is a phenomenon that has long intrigued ecologists. The effect was commonly thought to be an artifact of dimensionality reduction, and multiple techniques were developed to unravel this phenomenon and simplify interpretation. Here, we provide evidence that horseshoes arise as a consequence of distance metrics that saturate—a familiar concept in other fields but new to microbial ecology. This saturation property loses information about community dissimilarity, simply because it cannot discriminate between samples that do not share any common features. The phenomenon illuminates niche differentiation in microbial communities and indicates species turnover along environmental gradients. Here we propose a rationale for the observed horseshoe effect from multiple dimensionality reduction techniques applied to simulations, soil samples, and samples from postmortem mice. An in-depth understanding of this phenomenon allows targeting of niche differentiation patterns from high-level ordination plots, which can guide conventional statistical tools to pinpoint microbial niches along environmental gradients.

**IMPORTANCE** The horseshoe effect is often considered an artifact of dimensionality reduction. We show that this is not true in the case for microbiome data and that, in fact, horseshoes can help analysts discover microbial niches across environments.

## Abstract (precis)

The horseshoe effect is often considered an artifact of dimensionality reduction. We show that this is not true in the case for microbiome data and that, in fact, horseshoes can help analysts discover microbial niches across environments.

## OPINION/HYPOTHESIS

Ecological data sets, particularly those observed in microbiome studies, are typically sparse and high-dimensional, stymieing most conventional statistical techniques. Many numerical ecology software packages make use of distance-based statistics by calculating the distance between ecological communities to compare various ecosystems to each other over space and time. One of the most common exploratory analysis techniques is ordination, where the distances between the communities are embedded into a Euclidean space and are then visualized via principal-component analysis (PCA) (1). A widely used extension of this technique, where the distance metric can be varied, is called principal-coordinate analysis (PCoA) (1).

One phenomenon that commonly occurs in data sets containing ecological gradients is the horseshoe effect or Guttman effect (2). This phenomenon is typified by a linear gradient that appears as a curve in ordination space. The horseshoe effect, or its relative, the arch effect (where the ends of the gradient do not attract each other along the first principal coordinate as they do in the horseshoe effect), is observed using multiple types of ordinations, including principal-component analysis, principal-coordinate analysis, nonmetric multidimensional scaling, correspondence analysis, and many other methods (1). In 1982, the prevailing view of the horseshoe effect arose when it was described by Gauch as a mathematical artifact that obscures the underlying ecological gradient. Soon thereafter, detrending correspondence analysis (3) was invented to unbend the horseshoe using reciprocal averaging. Since then, detrending has become a practice commonly applied to ordinations in ecological data sets. Although these detrending techniques appear to provide a more intuitive visualization, they have been criticized as providing a distorted perspective of the underlying data, relying on many parameter settings that cannot be chosen in a principled way and obscuring true underlying patterns in the data (4).

In previous studies, it was shown that horseshoes can arise from band tables (5, 6). These tables consist of highly dense, nonzero values along the diagonal of the table and sparse values everywhere else. This pattern can be apparent when the rows and columns are sorted in the proper order. Although the idea that band tables lead to horseshoes is not a new idea, how this concept applies to microbial analyses is commonly misunderstood. Here we provide some insight into the mathematical structure of horseshoes.

**FIG 1:**
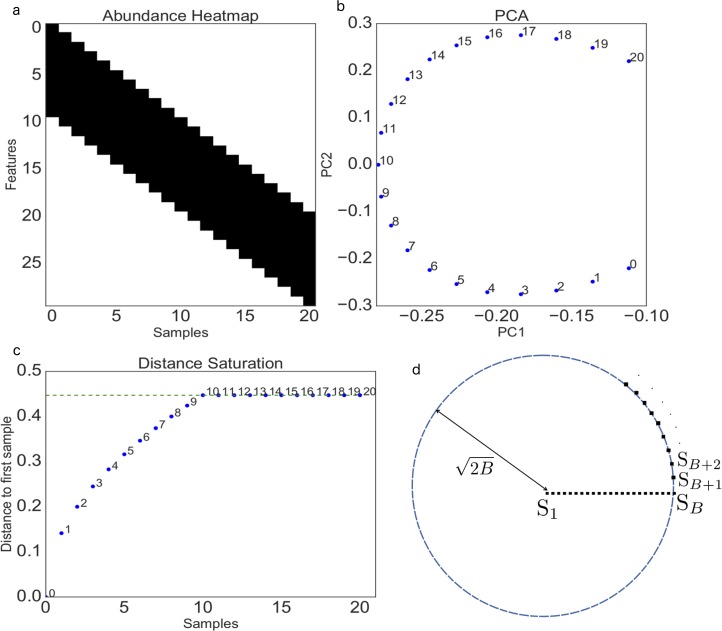
(a) A band table where the *y* axis data represent individual OTUs and the *x* axis data represent samples. Blocks that are colored black have a value of 1/10, while blocks that are colored white have a value of 0. (b) The first 2 components from a PCA of the band table, yielding the typical horseshoe shape. (c) The Euclidean distance from point 0 to all of the other points. (d) An illustration of the distance saturation property.

In Fig. 1a, we show a simulated band table where each vertical band is represented by a sample and contains 10 nonzero values. In typical microbiome data sets, these values could reflect operational taxonomic unit (OTU) or species counts; for simplicity, here we refer to them as species counts, although this concept can also be generalized to multiple data types, such as gene counts and metabolite abundances. Each sample in the table is shifted by 1 row, creating the band effect. When PCA is applied directly to this table, the first 2 eigenvectors yield a horseshoe pattern (Fig. 1b). Here, the band table is parameterized with a band size of 10, since each sample has exactly 10 nonzero values.

For closely local points, the Euclidean distance grows linearly along the gradient (Fig. 1c). However, after a certain point, the distance completely saturates. This property has been previously noted with Euclidean distance (3). The overlap of the first sample in the band table and sample 10 and beyond disappears, and the distance between these samples is maximized. This can yield unintuitive properties: sample 10 could be less dissimilar to sample 1 than sample 20. For instance, sample 10 could represent a medium-pH environment, sample 1 could represent a low-pH environment, and sample 20 could represent a high-pH environment. Sample 20 is expected to be more different from sample 1 than sample 10, since it contains very different microbes that thrive in high-pH environments. But as far as Euclidean distance is concerned, sample 10 is just as dissimilar to sample 1 as sample 20, just because there are no common bacteria shared between these samples. It is apparent that the saturation property of Euclidean distance does not capture all of the information about community dissimilarity along a gradient, simply because it cannot discriminate between samples that do not share any common features. Once the distance data are saturated, all samples that do not overlap lie within a ball of radius where *B* is the band size and the first point is the center of the ball, as shown in Fig. 1d.

**FIG 2:**
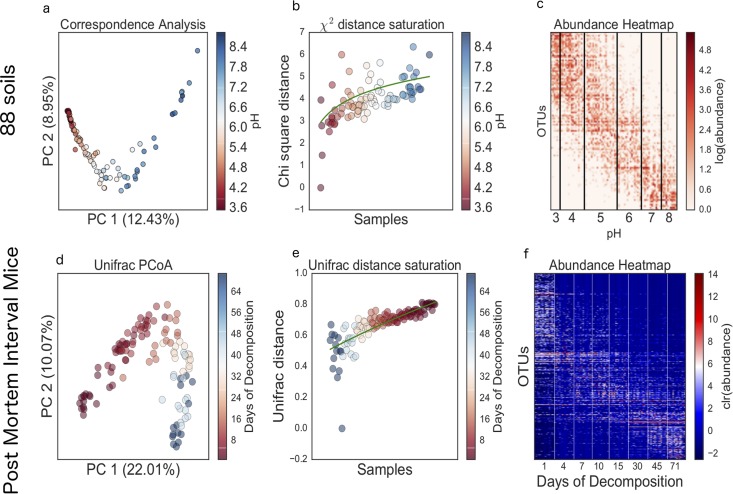
(a) Correspondence analysis of 88 soil samples. (b) Distance saturation of chi-squared metric, plotting the chi-squared distance of the first sample versus all of the other samples. (c) Heat map of log transformed OTU counts from the 88 soil samples with the samples sorted by pH and the OTUs sorted by mean pH. (d) PCoA of unweighted UniFrac distance. (e) UniFrac distance of a given sample from the last time point versus all of the samples. (f) Heat map of centered log ratio transformed (equation 2) OTU counts sorted by harvest days. clr, clearance.

This saturation property has been suggested in previous studies in other fields to give rise to horseshoes (5) and is an unintuitive property that can confound ecological interpretations if not understood properly. This property also restricts the possible trajectories of samples in the feature space, and gradients cannot be represented by linear trajectories in the real space (see Text S1 in the supplemental material). This means that communities in the original high-dimensional space do not arrange into linear trajectories in the first place and, when projected to lower dimensions, do not fall into linear trajectories. These trajectories are what we refer to as horseshoes. The horseshoe phenomenon is analogous to the familiar concept of saturation in molecular evolution, where two randomly evolving sequences saturate at 75% DNA sequence identity (assuming equal nucleotide frequencies), even if infinite time has elapsed (7). Consequently, distances that reflect a higher degree of molecular change need to be corrected for multiple substitutions in order to recover the molecular-clock-like behavior obtained in comparisons of more-similar sequences. This is why corrections according to models such as Jukes-Cantor or the Kimura 2-parameter model are required to obtain distances for reconstructing better phylogenetic trees. Analogous distance corrections are needed in microbial ecology for reconstructing better relationships among microbial communities (8).

10.1128/mSystems.00166-16.1 TEXT S1  A mathematical proof that *N* distinct samples separated by a minimum Euclidean distance cannot exist on a linear trajectory. Download TEXT S1, DOCX file, 0.3 MB. Copyright © 2017 Morton et al. 2017 Morton et al. This content is distributed under the terms of the Creative Commons Attribution 4.0 International license.

Note that horseshoes arise not only from PCA but from PCoA with a variety of distance metrics. Arch effects have plagued every multidimensional reduction technique that we have applied to a wide range of microbial ecology data sets (9). In the following case studies, we show that these distance metrics also have the saturation property. In addition, if a distance does not have this saturation property, there will not be an observed horseshoe artifact (see Fig. S1 in the supplemental material).

10.1128/mSystems.00166-16.2 FIG S1  (a) Description of the EMBAD metric, engineered to be nonsaturating. (b) A PCoA of the EMBAD applied to the 88 soil samples. (c) A PCoA of the EMBAD applied to the postmortem-interval mice. Download FIG S1, PDF file, 0.3 MB. Copyright © 2017 Morton et al. 2017 Morton et al. This content is distributed under the terms of the Creative Commons Attribution 4.0 International license.

### Case study 1—88 soil samples.

In this study, 88 soil samples were obtained from multiple locations across the United States having various levels of pH (10). The V4 region of the 16S rRNA gene (16S) within each organism was amplified and sequenced using 454 pyrosequencing to obtain relative abundances of microbial taxa. A matrix representing abundance values for each taxonomic unit per soil sample was used as input in correspondence analysis (CA) (11). The resulting ordination showed clear separation of the communities based on pH (Fig. 2a), which led to the same conclusion (that pH is a major driving factor in soil biogeography): i.e., pH has major impacts on the distribution of bacterial taxonomic units in soil (10). The CA represented in Fig. 2a also shows the classic horseshoe shape. Here we revisited that study, to better understand the horseshoe shape behind this data set.

To test the effect of another commonly used distance metric on the sample distribution, we analyzed the same soil data set applying the chi-squared distance test (Fig. 2b). Similarly to what was observed with Euclidean distance, which was applied in the simulation, the chi-squared distance increased sharply at pH 3 and 4 but began to saturate at a pH of 5. Also, when the OTU table was sorted by sample pH and mean pH of the OTUs (equation 1), the same band table pattern appeared, as we show in Fig. 1a. While the data at the diagonal are not completely dense, there are more nonzero values that are seen at the corners of the heat map. In line with the findings from the original study, this pattern is likely representative of niche differentiation of OTUs with respect to pH. The organisms that thrive in low-pH environments tend not to exist in high-pH environments and vice versa. Low-pH and high-pH samples are shown in Fig. 2c to have few overlapping species, a pattern not observed in the original study, as membership was evaluated at coarser levels of taxonomic resolution (10).

### Case study 2—postmortem mouse study.

In this study, 120 mice were sacrificed and allowed to decompose on soil. Mice were destructively sampled over approximately 8 weeks (12). 16S sequencing libraries were generated from total DNA extracted from swabs of the skin on the head, and relative abundance values were calculated for each bacterial OTU. A relative abundance matrix was generated for each library and used as input in PCA. This analysis generated a clear horseshoe (Fig. 2d) using unweighted UniFrac distance (13), with a gradient with respect to the time since death, possibly reflecting a changing skin microbiome during decomposition of the mouse carcass. When the samples were sorted by time since death using a strategy similar to that noted above, a band table emerged (Fig. 2f). Also, the unweighted UniFrac distance analysis appears to have the same saturation property as observed previously with Euclidean distance and chi-squared distance. Note that highest possible UniFrac distance is 1, suggesting that this distance metric can also be saturated. In Fig. 2b, while the distance has not completely saturated, these distances are quickly approaching the theoretical maximal UniFrac distance.

The striking changes in microbial communities during decomposition are associated with dramatic environmental biochemical changes, including increased pH, ammonia, and total nitrogen levels, all measured in the soil beneath the mice. Correspondingly, microbial communities are predicted to increase in the abundance of genes encoding proteins involved in important nitrogen cycling pathways such as amino acid degradation (e.g., glutamate dehydrogenase, lysine decarboxylase, and ornithine decarboxylase) and nitrate reduction (e.g., nitrate and nitrite reductase). Bacterial taxa in the families *Chromatiaceae* (OTU 46026 and 4482362) and *Rhizobiaceae* (OTU 4301099) are involved in nitrogen metabolism and become abundant as mouse bodies progress through the stages of decomposition (e.g., fresh, active decay, advanced decay). As shown in Fig. S1, all of these OTUs peak at specific time points. The two *Chromatiaceae* OTUs peak during active decay (bloating and purge of fluids) at 15 days of decomposition. The *Rhizobiaceae* OTU peaks during advanced decay (sinking and sagging flesh) at 30 days of decomposition and when pH, ammonia, and total nitrogen were measured at their highest levels (12).

To further validate whether saturation leads to the formation of horseshoes, a new distance metric, EMBAD (Earth Mover Band Aware Distance) was engineered to be nonsaturating. This distance metric uses prior knowledge about the ordering of the band table and is determined by calculating the flow between two samples. As shown in Fig. S1a, sample 1 and sample 2 have 4 species proportions each. To calculate the distance between sample 1 and sample 2, the probability mass of species 1 and species 2 needs to be shuffled over to species 3 and species 4. This concept is analogous to computing maximum flow along a pipe and can be calculated using Earth Mover’s distance metric (14).

For the 88 soil samples (Fig. S1b), the EMBAD was applied to the pH-sorted table. Therefore, even if two samples are not overlapping, samples closer together have a smaller distance value than samples further apart in the gradient. This is because the distance is defined to be not saturating and explicitly accounts for the pH gradient. The same strategy was employed for the postmortem-interval mice (Fig. S1c), sorting the table by decomposition days. The PCoA plots resulting from these applications of EMBAD suggest that a nonsaturating distance metric could remove the horseshoe effect from lower-dimensional projections of these abundances. This provides further evidence that this saturation property could explain the horseshoe phenomenon.

For the 88-soil-sample study, a permutational multivariate analysis of variance (PERMANOVA) was used to investigate the difference between soil samples with a pH less than 3 and soil samples with a pH greater than 8. With the EMBAD metric, the PERMANOVA gave a pseudo-F-statistic value of 650.5 and a *P* value of 0.0003, thus representing a much larger effect size than the original chi-squared distance metric, with a pseudo-F-statistic value of 3.8 and a *P* value of 0.0004 with 9,999 permutations. A similar trend was observed in the postmortem-interval mouse study in testing the interval from the first decomposition day to the last decomposition day using PERMANOVA. The EMBAD metric had a pseudo-F-statistic value of 439.8 and a *P* value of 0.0001 with 9,999 permutations, thus representing a larger effect size than the Unifrac distance metric, which had a pseudo-F-statistic value of 25.5 and a *P* value of 0.0001. This method is relieved from misinterpretations of data due to horseshoes and arches and facilitates the interpretation of taxonomic units along biologically significant gradients that reflect the selective pressure of these factors on the distribution of microbes.

The band patterns that we observed here are probably very common in ecology studies investigating species distribution patterns across spatial or temporal gradients. The pattern confirms microbial ecological fundamentals, i.e., that bacteria have acquired unique adaptations to the environment and occupy either a broad range of niches or very specific niches. In our case studies of the 88 soil samples—and of the postmortem mice—which we confirmed by using a band table pattern analysis approach, bacterial species showed different adaptations to pH and bacterial diversity changes over time during decomposition of mice carcasses. The band pattern approach that we applied here represents an additional method to visualize differences between microbial communities.

On the basis of our observations described here, the horseshoe effect appears in dimensionality reduction techniques due to the saturation property of distance metrics. While we have tested only a few distance metrics, it is suspected that a vast majority of these distance metrics exhibit the same property, which would also explain why horseshoes are encountered so frequently across many different fields. The saturation property has also been observed in multiple other fields, and other studies from different disciplines have led to similar conclusions (5). In spite of the saturation property of distance metrics, identifying horseshoes is still highly useful for identifying patterns concerning niche differentiation. These insights can ultimately guide additional statistical analyses, such as network analyses and indicator taxon analyses, to facilitate the targeted characterization of microbial niches.

### Methodology.

All analyses can be found under https://github.com/knightlab-analyses/horseshoe-analyses.

The mean gradient used for the 2 case studies was calculated as follows:

(1) $${\overset{¯}{g}}_{x}=\overset{N}{\underset{i=1}{\Sigma }}\;g_{i}\frac{x_{i}}{\Sigma_{j=1}^{D}x_{j}\;}$$

where *x*_*i*_ is the proportion of OTU *x* in sample *i*, ${\bar{g}}_{x}$ is the mean gradient of OTU *x*, and *g_i_* is the sample gradient at sample *i*. This calculation can be found in the gneiss package under the function mean_niche_estimator. The function used to sort the tables in Fig. 2c used niche_sort. In the 88-soil-sample study, the table was sorted by sample pH and mean pH of the samples in which the organisms were observed. In the postmortem mouse study, the table was sorted by the number of days of decomposition and the mean number of days that the organisms were observed in the samples.

The data represented in the heat map in Fig. 2f and the abundances in Fig. S2 were normalized using the center log ratio transformation given by the following equation:

10.1128/mSystems.00166-16.3 FIG S2  The center log ratio (equation 2) transformed abundances of *Rhizobiaceae* (OTU 4301099) and *Chromatiaceae* (OTU 46026 and OTU 4482362) versus time. Download FIG S2, PDF file, 0.1 MB. Copyright © 2017 Morton et al. 2017 Morton et al. This content is distributed under the terms of the Creative Commons Attribution 4.0 International license.

(2) $$clr(x)=[\ln\frac{x_{1}}{g\left(x\right)},\ldots ,\ln\frac{x_{D}}{g\left(x\right)}]=\ln x-\overset{¯}{\ln x}$$

where $g(x)=\sqrt[{n}]{\prod_{i=0}^{n}x_{i}}$ is the geometric mean and $\bar{\text{ln}x}=\text{ln}g(x)=\frac{1}{n}\overset{n}{\underset{i=0}{\Sigma }}$ is the average of the log transformed values. A pseudocount of 1 is added to all of the counts to prevent logarithms of zero occurring.

Analyses were performed using Scipy, Numpy, Matplotlib, Seaborn, Scikit-bio, and Gneiss.
