# Correction for Morton et al., “Uncovering the Horseshoe Effect in Microbial Analyses”

**DOI:** 10.1128/mSystems.00006-18

**Published:** 2018-02-06

**Authors:** James T. Morton, Liam Toran, Anna Edlund, Jessica L. Metcalf, Christian Lauber, Rob Knight

**Affiliations:** a Department of Computer Science, University of California, San Diego, La Jolla, California, USA; b Department of Pediatrics, University of California, San Diego, La Jolla, California, USA; c Department of Mathematics, École Normale Supérieure de Lyon, Lyon, France; d Genomic Medicine Group, J. Craig Venter Institute, La Jolla, California, USA; e Department of Animal Sciences, Colorado State University, Fort Collins, Colorado, USA; f Nestle Institute of Health Sciences, Lausanne, Switzerland

## AUTHOR CORRECTION

Volume 2, no. 1, e00166-16, 2017, https://doi.org/10.1128/mSystems.00166-16. Page 6, Acknowledgments, paragraph 1: the last sentence should read “Finally, we thank Susan Holmes for her insights on previous work understanding the horseshoe effect.”

