# Supplementary figures and images for: Uncovering the Horseshoe Effect in Microbial Analyses
Source: mSystems. 2017 Feb 21;2(1):e00166-16. doi: 10.1128/mSystems.00166-16 (PMC5320001; doi:10.1128/mSystems.00166-16)

**Supplemental material**

**Supplemental Proof 1**


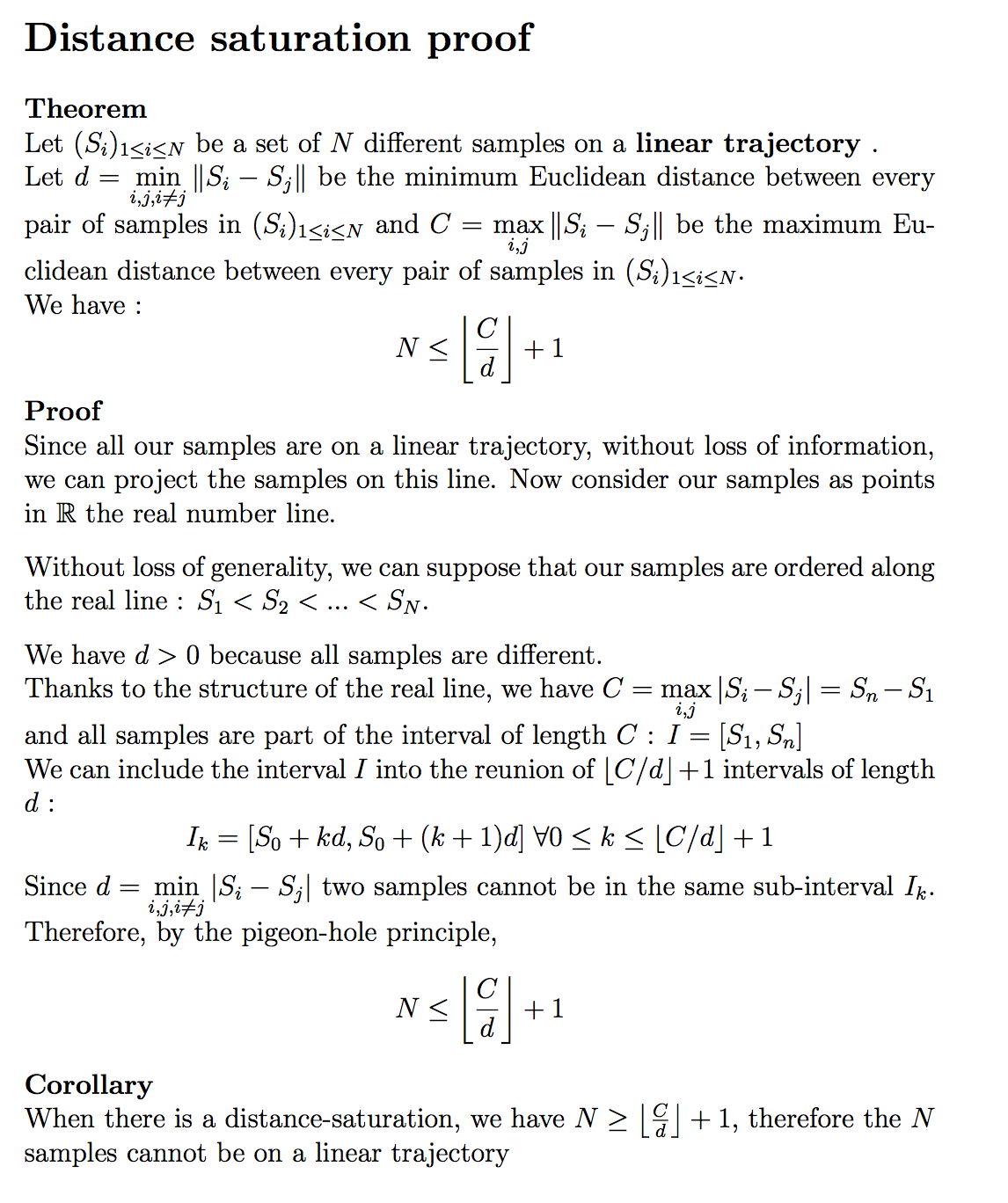

Supplement: TEXT S1 [file sys001172088s3.docx]

a

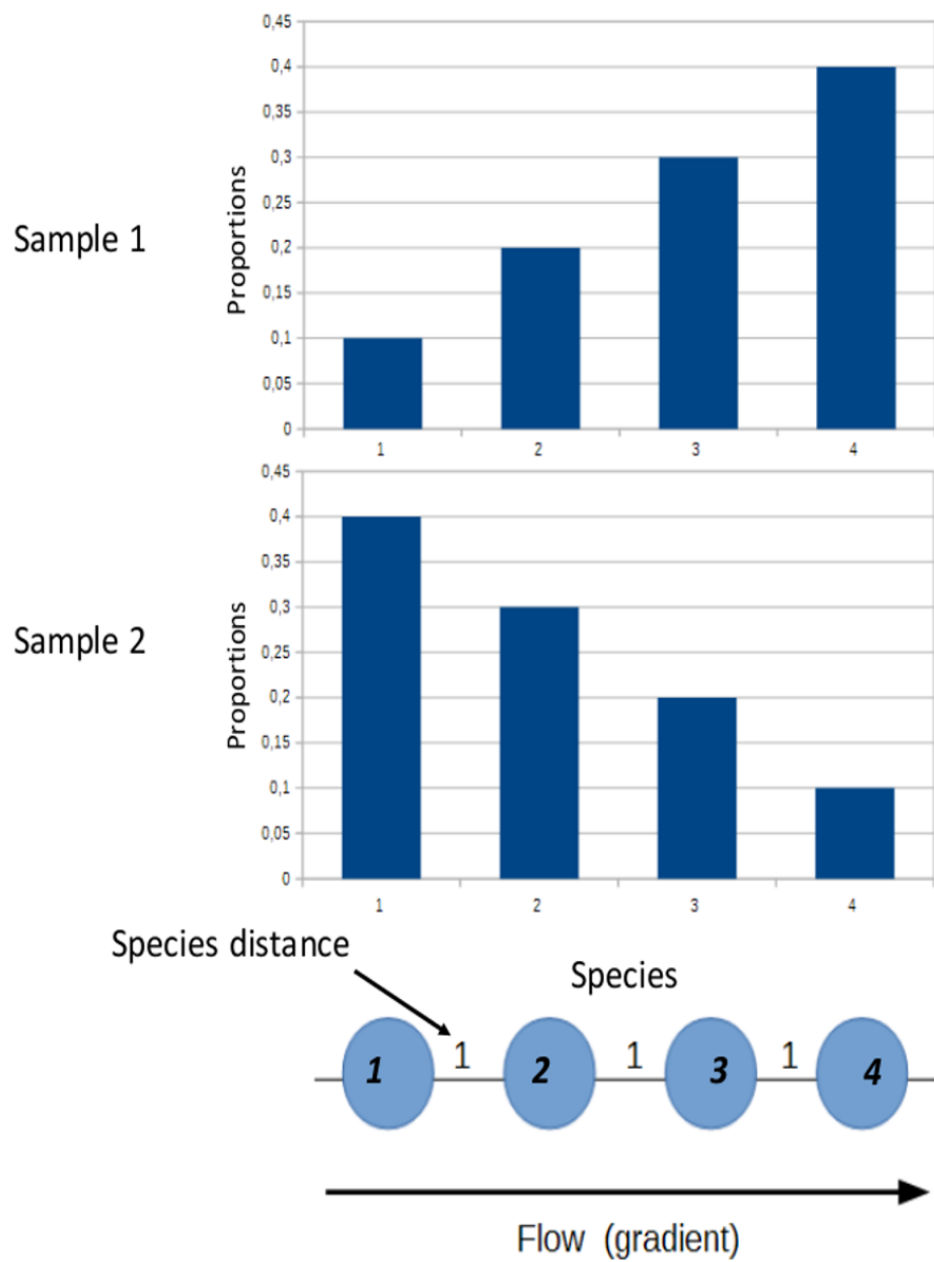

b

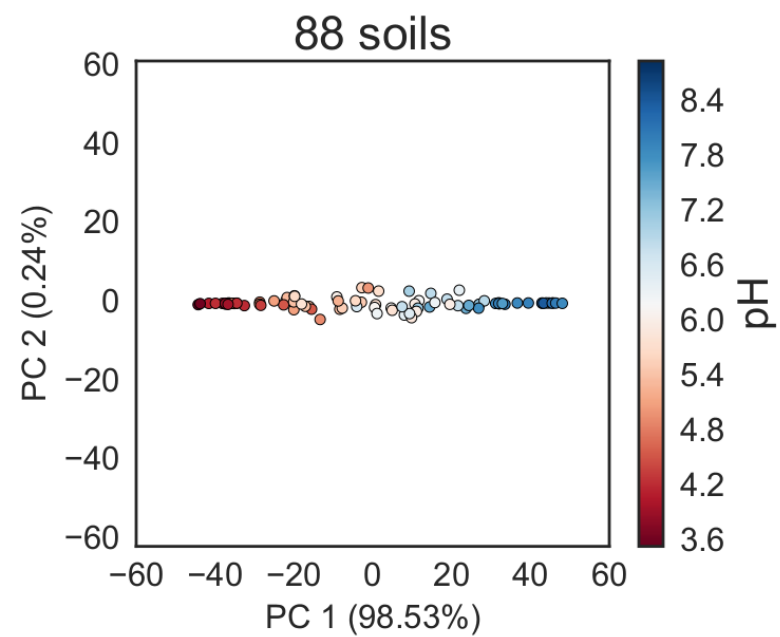

c

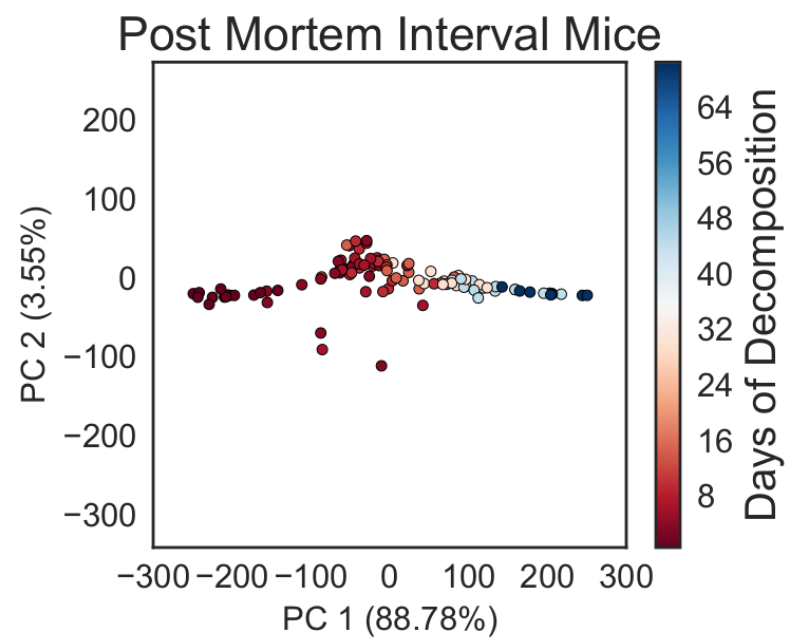

Supplement: FIG S1 [file sys001172088sf1.pdf]

OTU = 4301099

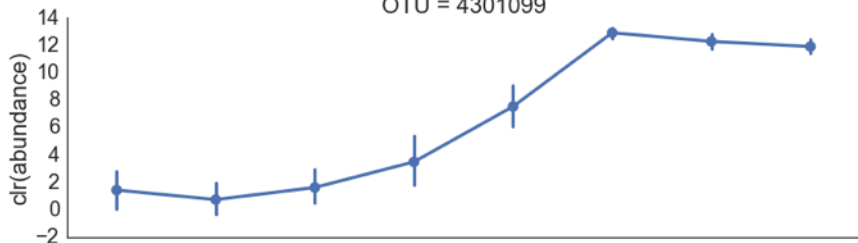

OTU = 4482362

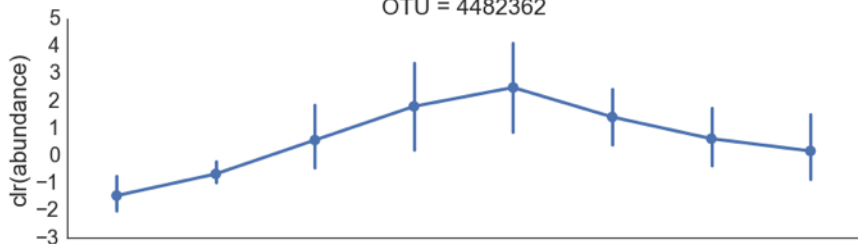

OTU = 46026

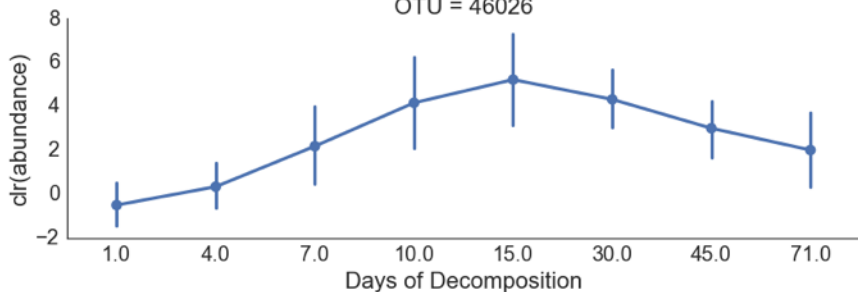

Supplement: FIG S2 [file sys001172088sf2.pdf]
